# Association between imbalanced lower extremity muscle quality and the development of knee osteoarthritis: a Systematic Review and meta-analysis

**DOI:** 10.3389/fphys.2026.1886678

**Published:** 2026-08-10

**Authors:** Zhi Liang, Guozhi Yang, Jiawen Zhang, Biao Tan, Yawei Dong, Yan Yan

**Affiliations:** 1 The Third Affiliated Hospital of Beijing University of Chinese Medicine, Beijing, China; 2 School of Orthopedics and Traumatology, Chongqing University of Chinese Medicine, Chongqing, China; 3 The First Affiliated Hospital of Chongqing University of Chinese Medicine, Chongqing, China; 4 Wangjing Hospital of China Academy of Chinese Medical Sciences, Beijing, China

**Keywords:** intermuscular adipose tissue, knee osteoarthritis, meta-analysis, muscle quantity, muscle quality, muscle strength, systematic review

## Abstract

**Background:**

Knee osteoarthritis (KOA) is the most prevalent musculoskeletal degenerative disease worldwide. Periarticular muscle abnormalities are considered closely associated with its pathogenesis, yet the relationships between various muscle parameters and KOA remain controversial.

**Objective:**

To clarify the correlations of thigh muscle quantity, muscle quality, and muscle strength with KOA through a systematic review and meta-analysis, and to provide evidence-based support for the risk screening and early intervention of KOA in high-risk populations.

**Methods:**

Relevant cross-sectional, cohort, and case-control studies published up to March 2026 were searched in PubMed, Embase, and MEDLINE (accessed via the Ovid). Methodological quality was assessed using the AHRQ scale for cross-sectional studies and the Newcastle-Ottawa Scale for cohort and case-control studies. Meta-analyses were performed using RevMan 5.3 software, and subgroup analyses stratified by study design were conducted to explore sources of heterogeneity.

**Results:**

A total of 24 studies involving 10,300 participants were included. Patients with KOA showed significantly increased cross-sectional areas of sartorius muscle (SMD = 0.09, *P* = 0.002) and flexor muscles (SMD = 0.09, *P* = 0.0008), and markedly reduced thickness of vastus lateralis muscle (SMD=-0.61, *P* = 0.03). Intermuscular adipose tissue content was significantly higher in KOA patients than in healthy controls (SMD = 0.35, *P* = 0.001). For muscle strength, KOA patients exhibited significant reductions in knee extensor torque (SMD=-1.09, *P* = 0.0001), extensor strength (SMD=-0.16, *P* = 0.0002), knee extensor relative strength (SMD=-0.15, *P* = 0.04), flexor torque (SMD=-0.95, *P* = 0.04), and flexor strength (SMD=-0.12, *P* = 0.004). Substantial heterogeneity was observed in key outcomes including knee extensor torque (I²=97%), knee flexor torque (I²=99%) and intermuscular adipose tissue (I²=72%). Low knee extensor strength was associated with a 1.45-fold increased odds of KOA (OR = 1.45, *P* = 0.01), and this association was more prominent in women (OR = 1.54, *P* = 0.0006).

**Conclusion:**

Alterations in thigh muscle quantity, quality and strength are significantly associated with KOA, particularly increased intermuscular adipose tissue infiltration and decreased knee extensor strength. Current evidence is predominantly derived from cross-sectional and case-control studies, and causal inferences cannot be established. These muscle parameters may serve as potential indicators for KOA risk stratification, and their predictive value and clinical utility require further verification in large-scale prospective cohort studies.

**Systematic Review Registration:**

https://www.crd.york.ac.uk/PROSPERO/view/CRD420261350323, identifier CRD420261350323.

## Introduction

Knee osteoarthritis (KOA) is one of the most common musculoskeletal diseases in the elderly population and a major cause of chronic pain, physical dysfunction, and reduced quality of life worldwide (GBD 2021 Osteoarthritis Collaborators, 2023; Lv et al., 2025). With global population aging and the increasing prevalence of obesity, the prevalence of this disabling disease is rising year by year in both elderly and young adults (Weng et al., 2024). Despite the considerable morbidity, economic burden, and social impact of KOA, no disease-modifying drugs are available to stop, slow, or reverse the progression of KOA (Duong et al., 2023). Current management strategies therefore emphasize symptom relief through non-pharmacological approaches such as exercise, education, and weight management (Lopez et al., 2026; Zhu et al., 2026). However, adherence to these interventions remains unsatisfactory, limiting their long-term efficacy (Farinelli et al., 2024). The benefits of exercise likely extend beyond symptom relief, as growing evidence indicates that physical activity can improve functional performance while modulating inflammatory pathways involved in osteoarthritis pathophysiology (Mauri et al., 2025). These findings further emphasize the importance of the musculoskeletal system in the progression and management of KOA. Patients with end-stage disease and persistent symptoms unresponsive to conservative treatment will eventually require joint replacement surgery (Anandacoomarasamy and March, 2010). Although our understanding of the pathogenesis of KOA continues to improve, there is still an urgent need to develop disease-modifying therapies and optimize early intervention strategies. Identifying modifiable associated factors and implementing optimal management is the core of preventing and treating KOA to reduce its global burden.

Knee osteoarthritis has long been regarded as a disease of articular cartilage, and degenerative loss of cartilage is considered its core pathological feature (Kerketta et al., 2026). However, recent evidence has shifted this paradigm and recognizes KOA as a complex whole-joint disease (Qiu et al., 2026) involving comprehensive changes in multiple joint structures, including cartilage, synovium, subchondral bone, meniscus, periarticular muscles, and supporting ligaments (Di Nicola, 2020; Coaccioli et al., 2022). Its structural and functional damage is closely related to knee alignment, knee instability, body weight, bone mineral density, quadriceps strength, and other factors (Felson et al., 1991; Zhang et al., 2000; Sharma et al., 2001). Within the research framework of whole-joint lesions, the role of periarticular muscles has received increasing attention. The quadriceps femoris and other thigh muscles buffer and disperse the mechanical load of the knee joint, and are crucial for maintaining dynamic joint stability and ensuring normal physiological movement patterns (Bennell et al., 2013). Decreased muscle quantity (defined as muscle volume and cross-sectional area, e.g., reduced muscle cross-sectional area) (Chen et al., 2024) and impaired muscle quality (defined as intramuscular and intermuscular adipose tissue infiltration, e.g., accumulation of intramuscular and intermuscular adipose tissue) (Pedroso et al., 2019) are considered to be closely associated with the onset and progression of knee osteoarthritis. However, current research conclusions remain controversial. Some studies have confirmed that insufficient muscle quantity or higher muscular fatty infiltration correlates with an increased risk of knee osteoarthritis and faster joint structural degeneration (Aily et al., 2025), whereas other studies have found no significant association (Jasinevicius et al., 2024). Notably, given that most existing evidence is derived from cross-sectional studies, it remains unclear whether these muscle abnormalities are causes or consequences of KOA; muscle changes may both contribute to joint degeneration and occur secondary to pain and reduced activity caused by KOA. In view of the inconsistent existing evidence, a comprehensive and systematic integrated analysis of available evidence is urgently needed.

Therefore, this study conducted a systematic review and meta-analysis to systematically synthesize existing evidence and comprehensively evaluate the associations between muscle quantity, muscle quality, and KOA. The results are expected to characterize periarticular muscle alterations in KOA, identify key muscle-related associated factors, and provide potential intervention targets for disease risk stratification and the formulation of early prevention and control strategies.

## Method

This study was registered with PROSPERO (registration number: CRD420261350323) and was conducted in accordance with the Preferred Reporting Items for Systematic Reviews and Meta-Analyses (PRISMA) guidelines (Moher et al., 2015).

### Search strategy and study selection

This study included cross-sectional, cohort, and case-control studies investigating the association between thigh muscles and the incidence of KOA published from inception until March 2026. The databases searched included PubMed, Embase, and MEDLINE (accessed via the Ovid). The search strategy employed Medical Subject Headings terms and relevant keywords to identify studies exploring the correlation between thigh muscles of different functions and the onset of KOA, covering indicators such as muscle strength, muscle cross-sectional area (CSA), intramuscular adipose tissue (intra-MAT) CSA, and their relationship with potential risk factors. The complete search strategies used for each database are detailed in **Supplementary Material 1**.

Two authors independently screened all titles and abstracts based on the inclusion and exclusion criteria. Disagreements at this stage were resolved by a third author. Subsequently, the full texts of potentially eligible studies were assessed for final inclusion. Any discrepancies arising throughout the process were resolved through consensus.

### Inclusion and exclusion criteria

#### Inclusion criteria

1. Published in English.
2. Cohort studies, case-control studies, and cross-sectional studies.
3. Study subjects are patients with a confirmed diagnosis of KOA or individuals from the general population assessed for incident KOA.
4. Reports should include the specific muscle indicators (muscle strength, muscle CSA, intra-MAT CSA), detailed measurement tools (e.g., MRI, CT, Ultrasound), as well as outcome indicators related to the incidence and imaging progression of knee OA.
5. Report potential risk factors and confounding factors.
6. Sufficient published data are available to estimate the hazard ratio (HR), relative risk (RR), odds ratio (OR), or weighted/standardized mean difference (WMD/SMD) and 95% confidence interval (CI).

#### Exclusion criteria

1. Secondary osteoarthritis associated with inflammatory diseases (such as rheumatoid arthritis), tumors, infections, or severe joint trauma.
2. Patients with a history of total knee arthroplasty (TKA) or those who have recently undergone major knee surgery.
3. Animal studies, biomechanical or *in vitro* biomarker studies, conference abstracts, and non-original research (e.g., editorials, literature reviews, commentaries, study protocols, and guidelines).
4. Studies with incomplete or irrecoverable outcome data, as well as duplicate data.

### Data extraction

This study extracted the following variables: first author, publication year, study location, study type, number of participants, diagnostic methods for KOA, and muscle image acquisition modalities. Meanwhile, a series of indicators reported in the included literature were collected for analysis, including muscle cross-sectional area, muscle volume, muscle thickness, intermuscular adipose tissue, muscle strength, and related risk factors.

Radiographic osteoarthritis was defined as an elevated Kellgren-Lawrence (KL) grade or a KL grade of 2 or higher (The Joint Surgery Branch of the Chinese Orthopaedic Association et al., 2021), which was adopted as the outcome indicator of this study. The mean values, standard deviations of each indicator, and ORs associated with KOA onset were extracted from each eligible study. According to the extracted ORs, the log odds ratio (logOR) and its corresponding standard error (SE) were calculated.

### Assessment of quality

For cross-sectional studies, the methodological quality of included literature was evaluated using an 11-item checklist recommended by the Agency for Healthcare Research and Quality (AHRQ) (Rostom et al., 2004). Each item was scored 0 for responses of “NO” or “UNCLEAR”, and 1 for a “YES” response. The overall quality of the studies was stratified according to the total score: low quality (0–3 points), moderate quality (4–7 points), and high quality (8–11 points).

For case-control and cohort studies, the Newcastle-Ottawa Scale (NOS) (Stang, 2010) was adopted to evaluate the methodological quality of included studies. The NOS comprises three core domains: Selection, Comparability, and Exposure. One star is granted for each qualified item. Specifically, each numbered item in the Selection and Exposure domains is limited to a maximum of one star, while up to two stars can be assigned for the Comparability domain.

In order to assess the quality of the meta-analysis results as a whole, a Grading of Recommendations Assessments, Development and Evaluation (GRADE) assessment was conducted meanwhile.

### Statistical analysis

This meta-analysis was performed using RevMan 5.3 software (The Cochrane Collaboration, London, UK). The mean values and standard deviations of each indicator were extracted from eligible original studies to systematically evaluate the correlations of muscle cross-sectional area, muscle volume, muscle thickness, intermuscular adipose tissue, muscle strength and other indicators with the occurrence and progression of KOA. Meanwhile, the adjusted ORs derived from multivariate regression models and their 95% CIs were collected. For studies that reported both unadjusted ORs and confounder-adjusted ORs, only the adjusted ORs were adopted to quantify the association between each indicator and the risk of KOA.

The I² statistic was calculated to assess the proportion of total variation attributable to inter-study heterogeneity. An I² value exceeding 50% indicates moderate to high heterogeneity. Given the heterogeneity in study designs, participant characteristics, and muscle measurement protocols across included studies, a random-effects model was employed for all analyses.

For studies that reported both unadjusted and multivariable-adjusted effect estimates, adjusted estimates were preferentially extracted to control for potential confounding. For studies reporting multiple independent datasets (e.g., stratified by sex), each dataset was included separately in the analysis; for studies reporting repeated measurements from the same population, only the most comprehensive baseline dataset was used to avoid double counting.

For any variable presenting with large heterogeneity, a sensitive analysis excluding outlier studies was conducted to investigate the potential sources of heterogeneity. Publication bias was assessed via funnel plots for outcomes with 10 or more study datasets.

## Results

### Literature search and screening

**Figure 1:**
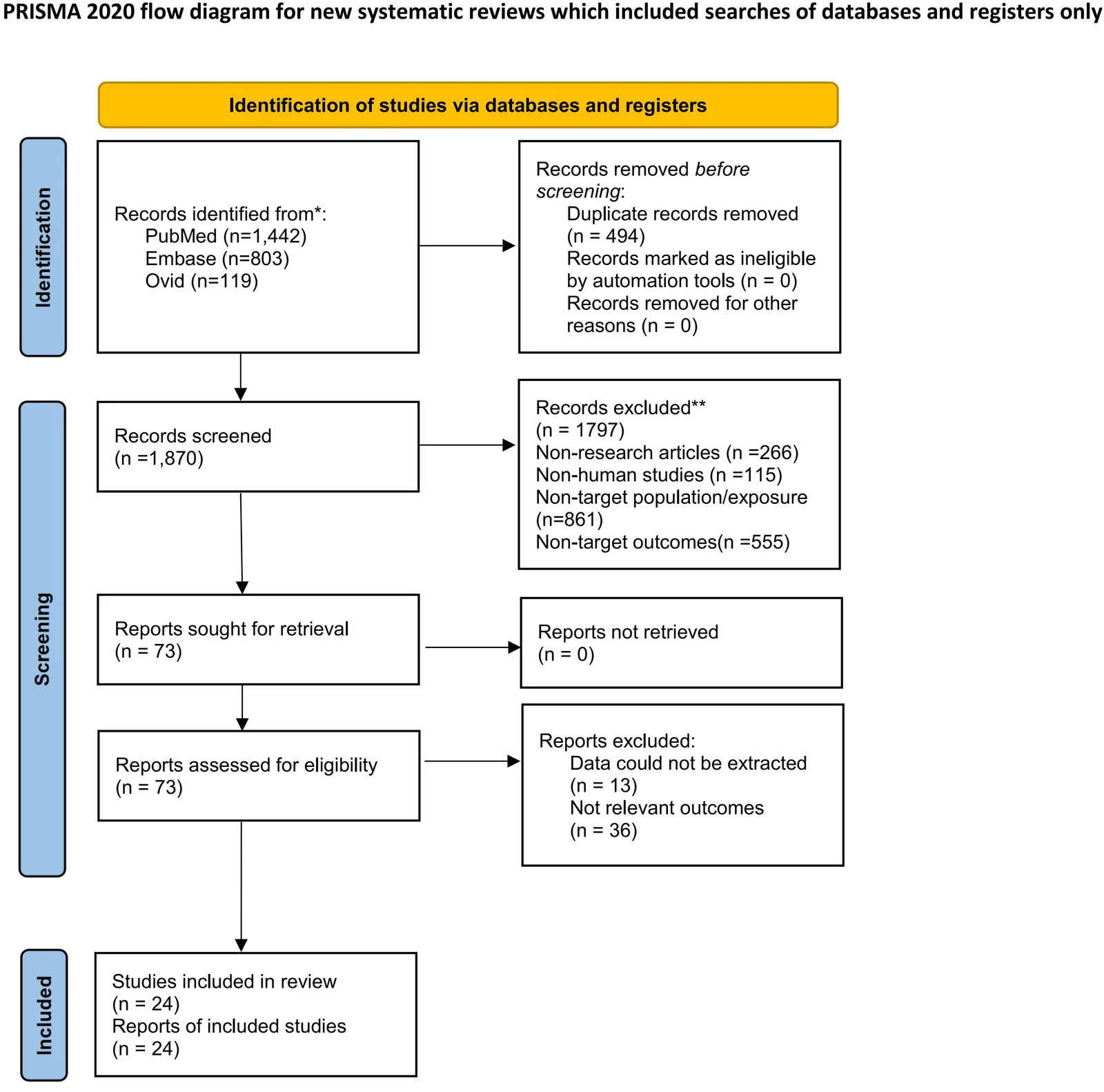
Flowchart of study selection.

From the electronic search conducted, 2,364 papers were identified from the three databases, in total, 24 papers (10,300 participants) were included in this review (**Figure 1**).

### Characteristics of the eligible studies

The 24 studies included in this review were derived from 8 different countries. The review comprised 11 case-control studies, 6 cohort studies, and 7 cross-sectional studies, with a total sample size of 10,300 participants. Variables extracted included muscle cross-sectional area, muscle volume, muscle thickness, intermuscular adipose tissue, muscle strength, and related risk factors. The main baseline characteristics of each study are summarized in **Table 1**.

**Table 1:** Characteristics of the included studies.

Reference	Region/year	Study design	Sample size(n)	Number of KOA(n)	Muscle image acquisition	Risk factors
Aily et al. (2025)	Brazil/2025	Cross-sectional	46	23	MRI	Intermuscular adipose tissue, Extensor strength, Knee extension torque
Aily et al. (2019)	Brazil/2019	Cross-sectional	40	20	Ultrasonography	Vastus Lateralis thickness, Vastus Lateralis thickness, Knee extension torque, Knee extension torque
Beattie et al. (2012)	Canada/2013	Case control	86	41	MRI	Quadriceps Femoris volume, Intermuscular adipose tissue
Chin et al. (2019)	Canada/2019	Cohort	163	20	MRI	Low knee extensor strength
Conroy et al. (2012)	USA/2012	Cross-sectional	425	334	MRI	Total thigh muscle Cross-sectional area, Quadriceps Femoris Cross-sectional, Intermuscular adipose tissue, Knee extensor relative strength
Culvenor et al. (2017)	Australia/2017	Case control	372	186	MRI	Flexors Cross-sectional area, Extensors Cross-sectional area, Extensor strength, Knee extensor relative strength, Flexor strength, Knee flexor relative strength, Low knee extensor strength
Culvenor et al. (2016)	Australia/2016	Case control	264	173	MRI	Knee extension torque, Knee flexion torque
Dannhauer et al. (2014)	Australia/2014	Cohort	86	43	MRI	Total thigh muscle Cross-sectional area, Adductor Cross-sectional area
Hislop et al. (2022)	Australia/2022	Cross-sectional	72	36	/	Knee extension torque
Jasinevicius et al. (2024)	Brazil/2024	Case control	80	40	DXA	Rectus Femoris Cross-sectional Area, Sartorius Cross-sectional area, Vastus Lateralis Cross-sectional area
Kemnitz et al. (2017)	Australia/2017	Cohort	1016	284	/	Knee extension torque, Knee flexion torque
Kumar et al. (2014)	USA/2014	Case control	96	30	MRI	Intermuscular adipose tissue, Knee extension torque
Maly et al. (2013)	Canada/2013	Case control	125	73	MRI	Quadriceps Femoris volume, Intermuscular adipose tissue
Mohajer et al. (2022)	Germany/2022	Cohort	4634	2317	MRI	Flexors Cross-sectional area, Total thigh muscle Cross-sectional area, Sartorius Cross-sectional area, Quadriceps Femoris Cross-sectional, Adductor Cross-sectional area, Intermuscular adipose tissue
Ruhdorfer et al. (2014)	Australia/2014	Case control	110	55	MRI	Vastus Medialis Cross-sectional Area, Rectus Femoris Cross-sectional Area, Vastus Lateralis Cross-sectional area, Quadriceps Femoris Cross-sectional, Adductor Cross-sectional area, Extensor strength, Knee extensor relative strength, Flexor strength, Knee flexor relative strength
Segal et al. (2010)	USA/2010	Cohort	2182	576	MRI	Extensor strength, Flexor strength, Low knee extensor strength, Low knee extensor strength
Takagi et al. (2018)	Japan/2018	Cohort	211	86	/	Low knee extensor strength
Taniguchi et al. (2023)	Japan/2023	Case control	50	19	MRI	Quadriceps Femoris volume
Taniguchi et al. (2015)	Japan/2015	Case control	31	13	Ultrasonography	Vastus Lateralis thickness
Teoli et al. (2022)	Germany/2022	Cross-sectional	44	22	MRI	Vastus Medialis Cross-sectiona, Area1 Knee extension torque
Watabe et al. (2025)	Japan/2025	Case control	30	15	CT	Vastus Medialis Cross-sectional Area
Yamauchi et al. (2020)	Japan/2020	Cross-sectional	40	20	MRI	Quadriceps Femoris volume
Yamauchi et al. (2019)	Japan/2019	Cross-sectional	50	27	MRI	FlexorsCross-sectional area, Total thigh muscle Cross-sectional area, Extensors Cross-sectional area, Adductor Cross-sectional area, Intermuscular adipose tissue
Zhang et al. (2020)	China/2020	Case control	47	25	DXA	Extensor strength, Flexor strength, Knee extension torque, Knee flexion torque

### Assessment of quality

11 case-control studies, 6 cohort studies and 7 cross-sectional studies were included in the 24 papers of this review. The average quality score of all included case-control studies was 5.8 points, that of cohort studies was 7 points, and that of cross-sectional studies was 6.4 points. **Supplementary Material 2**: **Supplementary Tables 1**-**3** present the detailed evaluation results.

The overall certainty of evidence for main outcomes was rated as low to moderate according to GRADE criteria, mainly downgraded due to observational study design and high heterogeneity in some outcomes (**Supplementary Material 3**).

The most common methodological limitations across included studies were insufficient control for confounding factors (e.g., BMI, age), unreported non-response rates, and inconsistent KOA diagnostic criteria. These limitations may introduce selection bias and measurement bias.

### Meta-analysis

#### Sartorius cross-sectional area

**Figure 2:**
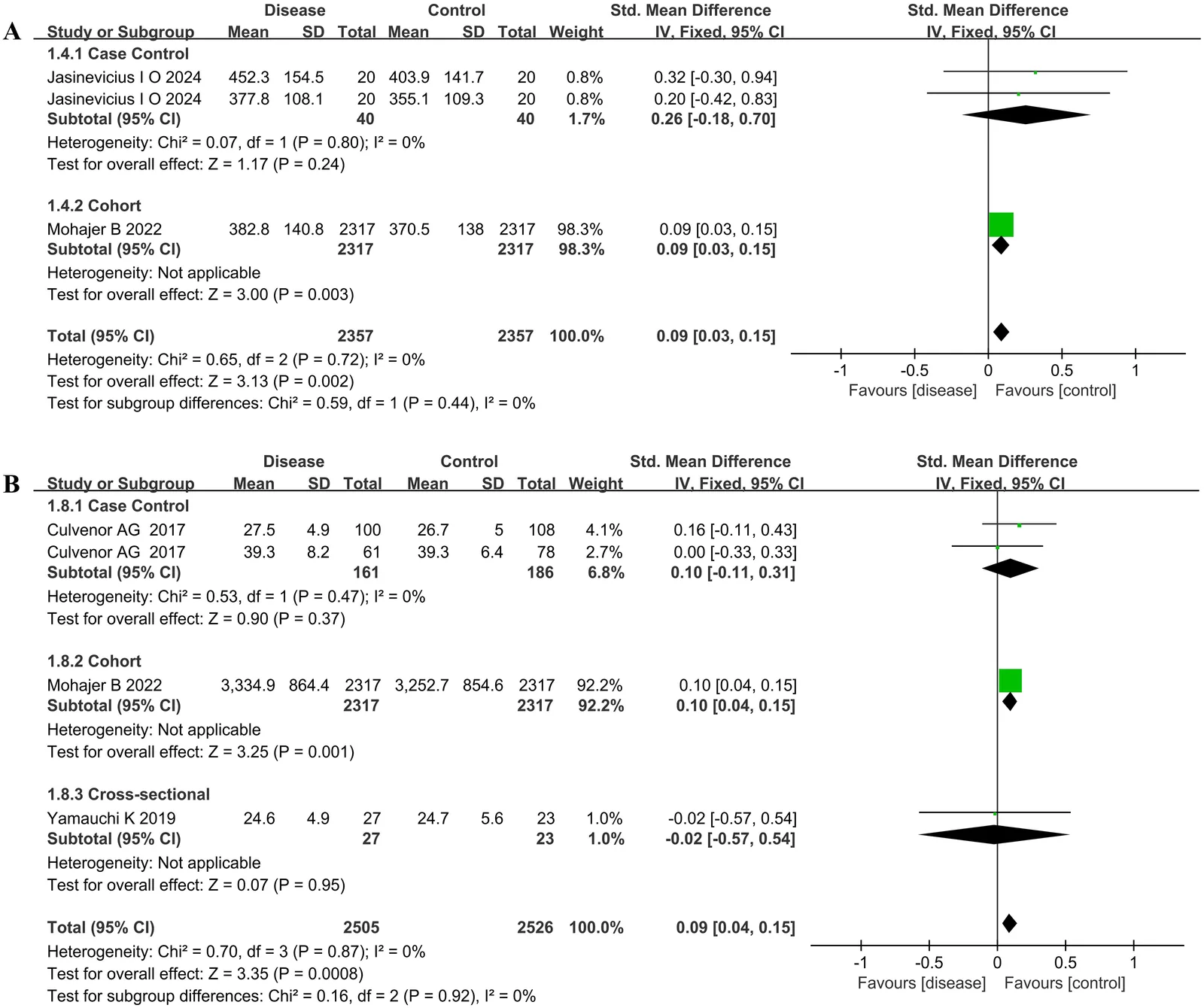
Forest plot: **(A)** sartorius cross-sectional area; **(B)** flexors cross-sectional area.

Two studies (Mohajer et al., 2022; Jasinevicius et al., 2024) with 3 datasets investigated the difference in sartorius CSA between patients with KOA and healthy individuals. The results showed that the sartorius CSA was significantly higher in KOA patients than in controls [I²=0%, SMD = 0.09 (0.03, 0.15), *P* = 0.002]. However, the effect size was very small, suggesting limited clinical significance of this morphological change alone. The subgroup analysis results suggest that the sartorius CSA did not demonstrate statistical significance in the meta-analysis of case-control studies (**Figure 2A**).

#### Flexors cross-sectional area

Three studies (Culvenor et al., 2017; Yamauchi et al., 2019; Mohajer et al., 2022) with 4 datasets explored the differences in flexor CSA between patients with KOA and healthy individuals. The results demonstrated that the flexor CSA was significantly larger in patients with KOA than in controls [I²=0%, SMD = 0.09 (0.04, 0.15), *P* = 0.0008]. However, the effect size was very small, indicating that this change alone has limited clinical relevance. The subgroup analysis results suggest that the flexor CSA did not demonstrate statistical significance in the meta-analysis of case-control studies or cross-sectional study (**Figure 2B**).

#### Vastus lateralis thickness

**Figure 3:**
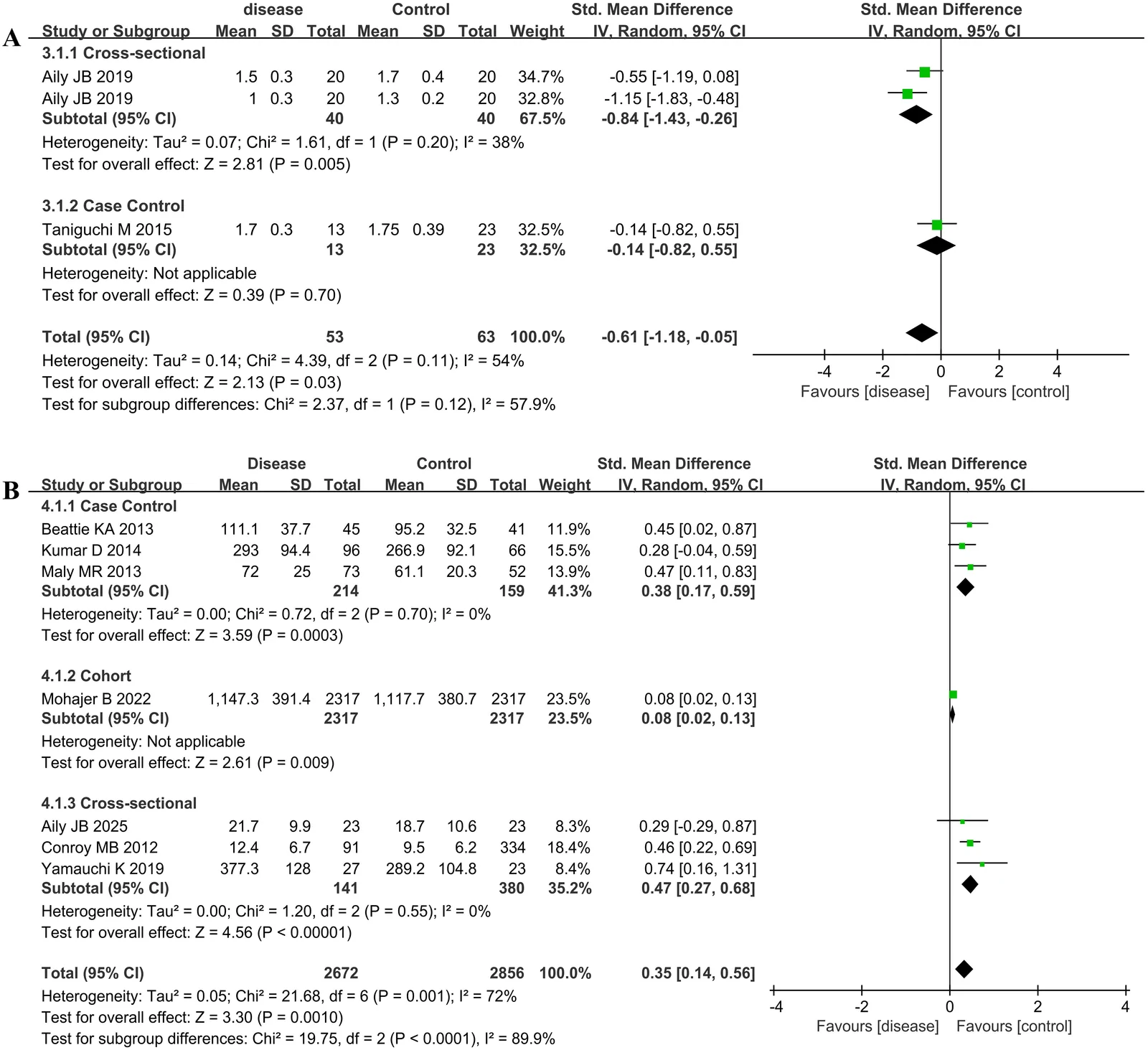
Forest plot: **(A)** vastus lateralis thickness; **(B)** intermuscular adipose tissue.

Two studies (Taniguchi et al., 2015; Aily et al., 2019) with 3 datasets investigated the difference in vastus lateralis thickness between patients with KOA and healthy individuals. The results showed that vastus lateralis thickness was significantly lower in patients with KOA than in healthy individuals [I²=54%, SMD=-0.61 (95% CI: -1.18, -0.05), *P* = 0.03], with a statistically significant difference. But the subgroup analysis results suggest that the vastus lateralis thickness did not demonstrate statistical significance in the meta-analysis of case-control study (**Figure 3A**).

#### Intermuscular adipose tissue

Six studies (Beattie et al., 2012; Conroy et al., 2012; Maly et al., 2013; Kumar et al., 2014; Yamauchi et al., 2019; Mohajer et al., 2022; Aily et al., 2025) investigated the difference in intermuscular adipose tissue between patients with KOA and healthy individuals. The results demonstrated that intermuscular adipose tissue was significantly higher in patients with KOA than in healthy individuals [I²=72%, SMD = 0.35 (95% CI: 0.14, 0.56), *P* = 0.001], and the difference was statistically significant. The subgroup analysis suggested that the between-group differences in intermuscular adipose tissue were statistically significant in case-control studies, cohort study and cross-sectional studies alike. (**Figure 3B**).

#### Knee extensor torque

**Figure 4:**
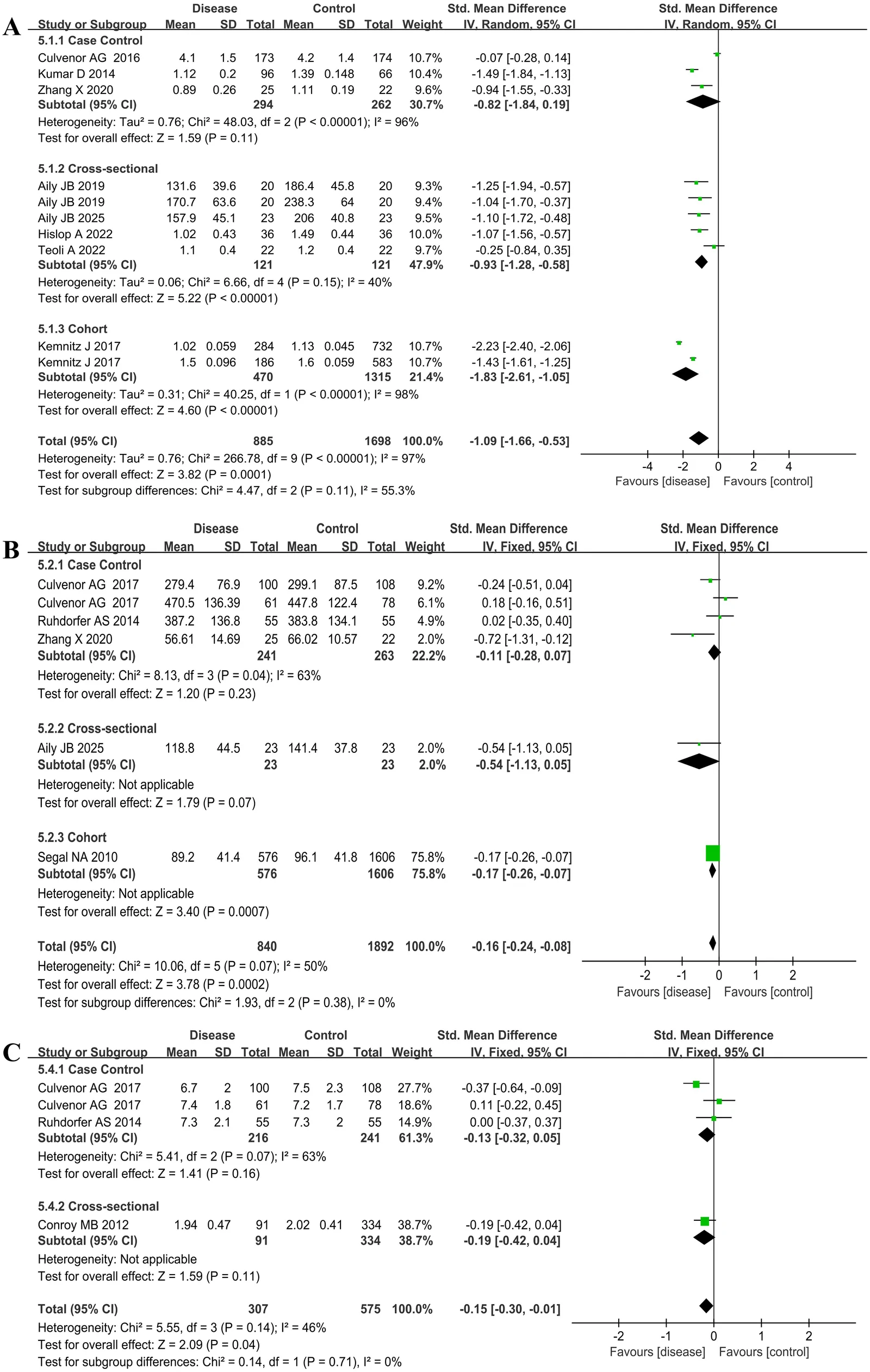
Forest plot: **(A)** knee extensor torque; **(B)** extensor strength; **(C)** knee extensor relative strength.

Eight studies (Kumar et al., 2014; Culvenor et al., 2016; Kemnitz et al., 2017; Aily et al., 2019; Zhang et al., 2020; Hislop et al., 2022; Teoli et al., 2022; Aily et al., 2025) involving 10 datasets analyzed the difference in knee extensor torque between KOA patients and healthy individuals. The results indicated that knee extensor torque was significantly lower in KOA patients [I²=97%, SMD=−1.09 (95% CI: −1.66, −0.53), *P* = 0.0001], with a statistically significant difference. The subgroup analysis suggested that the between-group differences in knee extensor torque were statistically significant in case-control studies, cohort study and cross-sectional studies alike (**Figure 4A**).

#### Extensor strength

Five studies (Segal et al., 2010; Ruhdorfer et al., 2014; Culvenor et al., 2017; Zhang et al., 2020; Aily et al., 2025) with 6 datasets analyzed the differences in extensor strength between patients with KOA and healthy individuals. The results showed that extensor strength was significantly lower in KOA patients than in healthy individuals [I^2^ = 50%, SMD=-0.16 (95% CI: -0.24, -0.08), *P* = 0.0002], with a statistically significant difference. But the subgroup analysis results suggest that the extensor strength did not demonstrate statistical significance in the meta-analysis of case-control studies or cohort study (**Figure 4B**).

#### Knee extensor relative strength

Three studies (Conroy et al., 2012; Ruhdorfer et al., 2014; Culvenor et al., 2017) containing 4 datasets explored the difference in relative knee extensor strength between KOA patients and healthy individuals. The results revealed that relative knee extensor strength was significantly lower in patients with KOA than in healthy individuals[I²=46%, SMD=-0.15 (95% CI: -0.30, -0.01), *P* = 0.04], with a statistically significant difference. But the subgroup analysis results suggest that the knee extensor relative strength did not demonstrate statistical significance in the meta-analysis of case-control studies or cross-sectional study (**Figure 4C**).

#### Knee flexion torque

**Figure 5:**
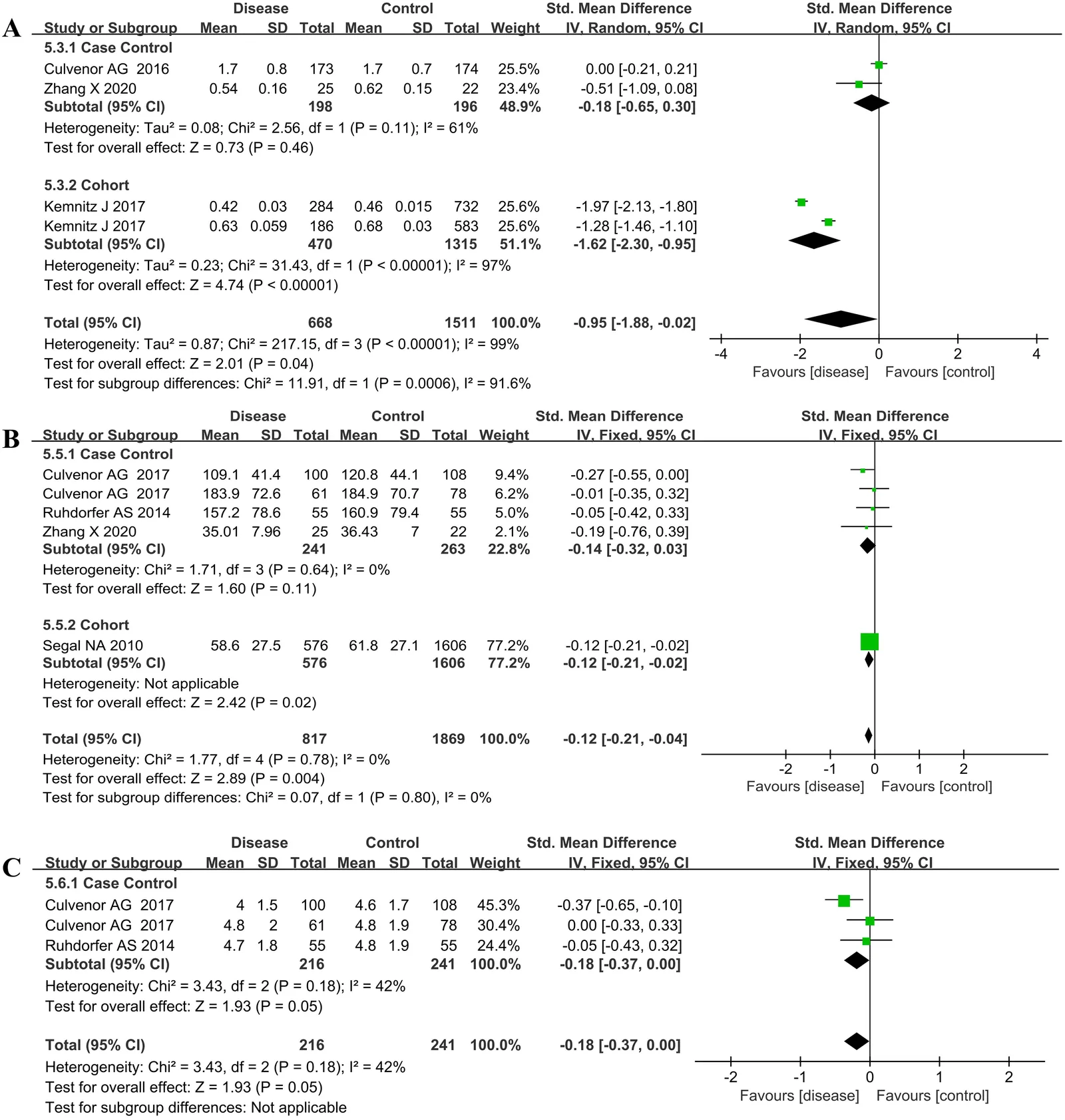
Forest plot: **(A)** knee flexion torque; **(B)** flexor strength; **(C)** knee flexor relative strength.

Three studies (Culvenor et al., 2016; Kemnitz et al., 2017; Zhang et al., 2020) comprising 4 datasets analyzed the differences in knee flexor torque between patients with KOA and healthy individuals. The results indicated that knee flexor torque was significantly lower in KOA patients [I²=99%, SMD=-0.95 (95% CI: -1.88, -0.02), *P* = 0.04], and the difference was statistically significant. But the subgroup analysis results suggest that the knee flexion torque did not demonstrate statistical significance in the meta-analysis of case-control studies (**Figure 5A**).

#### Flexor strength

Four studies (Segal et al., 2010; Ruhdorfer et al., 2014; Culvenor et al., 2017; Zhang et al., 2020) involving 5 datasets explored the difference in knee flexor strength between KOA patients and healthy individuals. The results showed that knee flexor strength was significantly lower in patients with KOA than in healthy individuals[I²=0%, SMD=-0.12 (95% CI: -0.21, -0.04), P = 0.004], with a statistically significant difference. But the subgroup analysis results suggest that the flexor strength did not demonstrate statistical significance in the meta-analysis of case-control studies (**Figure 5B**).

#### Knee flexor relative strength

Two studies (Ruhdorfer et al., 2014; Culvenor et al., 2017) with 3 datasets analyzed the difference in relative knee flexor strength between KOA patients and healthy individuals. The results revealed that relative knee flexor strength was significantly lower in KOA patients [I²=42%, SMD=-0.18 (95% CI: -0.37, 0.00), *P* = 0.05] (**Figure 5C**).

#### Low knee extensor strength

**Figure 6:**
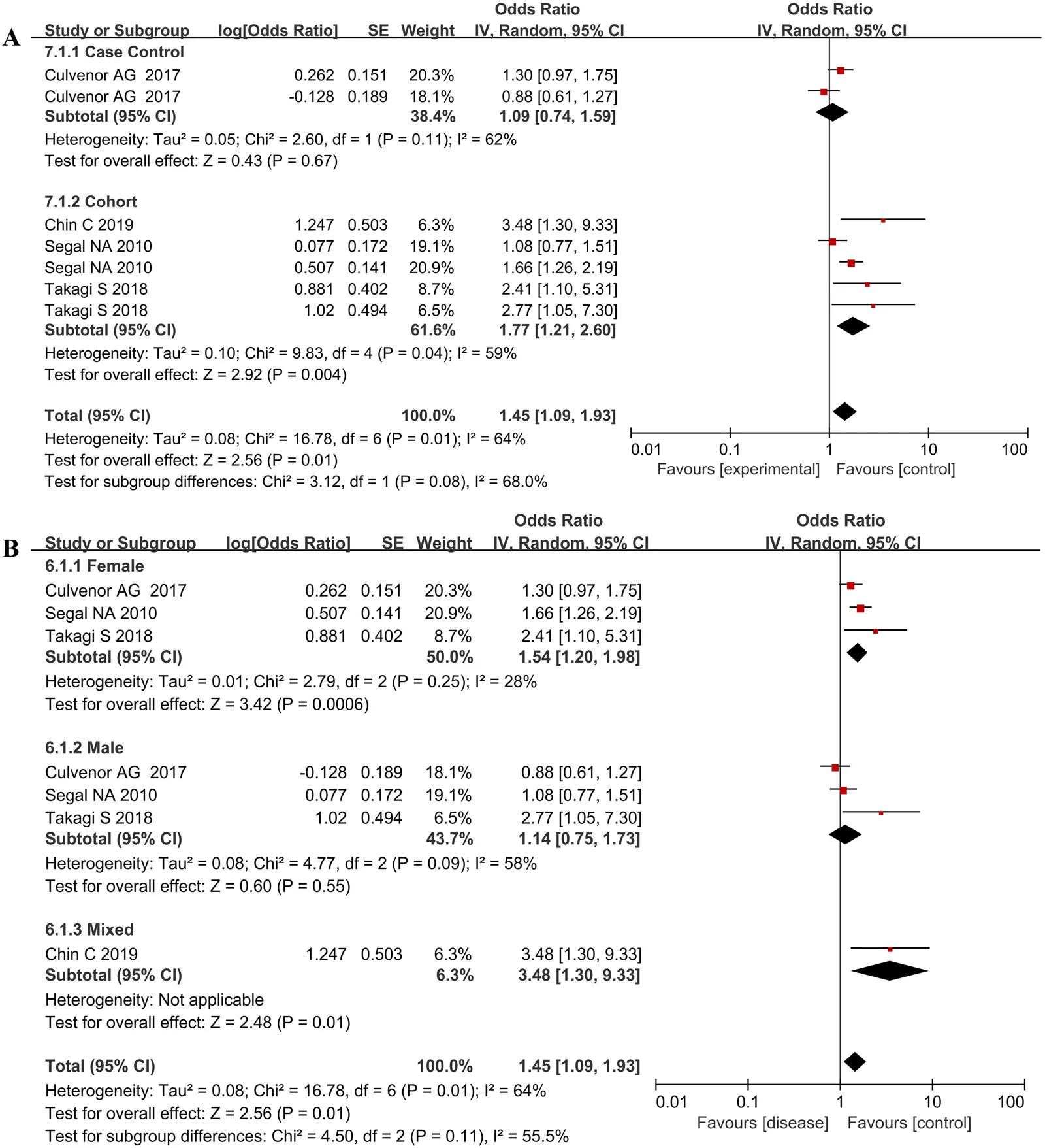
Forest plot: **(A)** low knee extensor strength in subgroups stratified stratified by different study types; **(B)** low knee extensor strength in subgroups stratified stratified by gender;.

Four studies (Segal et al., 2010; Culvenor et al., 2017; Takagi et al., 2018; Chin et al., 2019) explored the association between low knee extensor strength and KOA. The pooled results showed that low knee extensor strength was associated with a 1.45-fold higher odds of KOA [I²=64%, OR = 1.45 (95% CI: 1.09, 1.93), *P* = 0.01]. But the results of subgroup analysis indicated that the meta-analysis of case-control studies failed to confirm that low knee extensor strength is a risk factor for KOA (**Figure 6A**). Subgroup analysis indicated that low knee extensor strength was associated with a 1.54-fold higher odds of KOA in females[I²=28%, OR = 1.54 (95% CI: 1.20, 1.98), *P* = 0.0006], while no statistically significant difference was observed in the male subgroup[I²=58%, OR = 1.14 (95% CI: 0.75, 1.73), *P* = 0.55]. In the unstratified subgroup without gender distinction, low knee extensor strength was linked to a 3.48-fold higher risk of KOA[OR = 3.48 (95% CI: 1.30, 9.33), *P* = 0.01] (**Figure 6B**).

#### Other factors

We further performed meta-analyses on other indicators, including the cross-sectional areas of the rectus femoris (Ruhdorfer et al., 2014; Jasinevicius et al., 2024) (**Supplementary Figure 1A**), vastus lateralis (Ruhdorfer et al., 2014; Jasinevicius et al., 2024; Watabe et al., 2025) (**Supplementary Figure 1B**), vastus medialis (Ruhdorfer et al., 2014; Teoli et al., 2022; Watabe et al., 2025) (**Supplementary Figure 1C**), adductor muscles (Dannhauer et al., 2014; Ruhdorfer et al., 2014; Yamauchi et al., 2019; Mohajer et al., 2022) (**Supplementary Figure 1D**), total thigh muscle (Conroy et al., 2012; Dannhauer et al., 2014; Yamauchi et al., 2019; Mohajer et al., 2022) (**Supplementary Figure 1E**), quadriceps femoris (Conroy et al., 2012; Ruhdorfer et al., 2014; Mohajer et al., 2022) (**Supplementary Figure 1F**), and extensor muscles (Culvenor et al., 2017; Yamauchi et al., 2019; Mohajer et al., 2022) (**Supplementary Figure 1G**), as well as quadriceps femoris volume (Beattie et al., 2012; Maly et al., 2013; Yamauchi et al., 2020; Taniguchi et al., 2023) (**Supplementary Figure 1H**). No significant differences in these indicators were identified between KOA patients and healthy individuals in the present meta-analysis.

### Sensitivity analysis and publication bias

We conducted a sensitivity analysis using the exclusion method for four indicators with high heterogeneity, namely intermuscular adipose tissue, knee extensor torque, knee flexion strength, and low knee extensor strength. The results showed that after excluding the study with the largest weight for intermuscular adipose tissue, the heterogeneity decreased to 0%, while the results of the remaining indicators remained stable (**Supplementary Material 2**: **Supplementary Table 4**).

Sensitivity analyses after excluding low-quality studies revealed that the pooled effect sizes of the four indicators with high heterogeneity remained stable, indicating that the main conclusions were not substantially influenced by low-quality studies. Detailed literature quality assessment results and sensitivity analysis data are provided in **Supplementary Material 2**: **Supplementary Table 5**.

Funnel plot analysis was only performed for knee extensor torque (10 studies), which was the only outcome with ≥10 included study datasets. The funnel plot was approximately symmetrical, suggesting no obvious publication bias for this outcome (**Supplementary Figure 2**). For all other outcomes with fewer than 10 studies, funnel plot analysis was not performed due to insufficient statistical power, and potential publication bias cannot be excluded.

## Discussion

KOA ranks as the leading musculoskeletal disorder responsible for chronic pain and physical dysfunction among the elderly worldwide. The understanding of its pathogenesis has evolved from simple cartilage degeneration to a comprehensive perspective of whole-joint disease (Di Nicola, 2020; Coaccioli et al., 2022; Qiu et al., 2026). Accumulating attention has been paid to the pivotal role of periarticular muscles in maintaining knee dynamic stability and buffering mechanical loading (Bennell et al., 2013). Nevertheless, previous studies have yielded inconsistent conclusions regarding the associations between thigh muscle characteristics and KOA. The independent effects of muscle quantity, muscle quality and muscle strength on the onset and progression of KOA remain unclear, which directly hinders the formulation of early prevention strategies for KOA targeting muscle-related interventions. The present systematic review and meta-analysis integrated data from 24 studies involving 10,300 participants, comprehensively exploring the correlations of multiple dimensional indicators (including thigh muscle cross-sectional area, thickness, intermuscular adipose tissue content and muscle strength) with KOA, thereby providing high-quality evidence for clarifying the role of muscles in the pathological process of KOA.

The current results demonstrated that KOA patients exhibited significantly larger cross-sectional areas of the sartorius and flexor muscles, as well as markedly reduced vastus lateralis thickness compared with healthy individuals. No significant intergroup differences were observed for most other indicators, including the cross-sectional areas of the rectus femoris, vastus lateralis, vastus medialis, adductor muscles and total thigh muscle mass. These findings partially explain the discrepancies in previous literature: heterogeneous selection of muscle subgroups across studies may lead to contradictory outcomes. The increased CSA of sartorius and flexor muscles may represent compensatory hypertrophy in response to quadriceps dysfunction, which is a plausible mechanistic hypothesis. Previous research has highlighted the importance of muscular coordination, proposing that the synergistic activation pattern dominated by the hamstrings and quadriceps can effectively generate corresponding knee flexion and extension torques, jointly supporting the knee joint against external loads (Flaxman et al., 2021). When the function of primary knee extensors such as the quadriceps is impaired, synergistic muscles including the sartorius and hamstrings (especially the biceps femoris) are activated to compensate for maintaining knee dynamic stability (Shelburne et al., 2005), resulting in a compensatory increase in their cross-sectional areas. However, this hypothesis cannot be directly verified by the predominantly evidence included in this review, and longitudinal studies are needed to confirm the temporal sequence of these muscle changes.

As a crucial component of the quadriceps, the vastus lateralis accounts for approximately 34% of the total physiological cross-sectional area of the quadriceps (Bakenecker et al., 2019). Its remarkable thickness reduction indicates obvious muscle atrophy in KOA patients, which is closely related to the essential role of the vastus lateralis in knee extension and its high sensitivity to altered mechanical loading (Sharifnezhad et al., 2014). Notably, no significant association was identified between total thigh muscle cross-sectional area and KOA in this meta-analysis, indicating that simple changes in muscle volume are not the core risk factor for KOA pathogenesis; alterations in muscle quality and function may be more important than muscle quantity alone.

This study confirmed a significant association between intermuscular adipose tissue content and KOA. Intermuscular adipose tissue content was significantly higher in KOA patients than in healthy controls (SMD = 0.35, *P* = 0.001), and this association remained stable even after excluding the study with the largest weight. This finding further validates that increased intermuscular adipose tissue is an important feature associated with KOA, and may be involved in the pathological process of the disease through inflammatory and mechanical pathways. Intermuscular fat infiltration is not merely simple fat accumulation but a metabolic disorder. Ectopically deposited adipocytes secrete abundant pro-inflammatory cytokines and adipokines (Yan et al., 2026), inducing muscular insulin resistance and reducing muscle contraction efficiency via paracrine pathways (Gui et al., 2025). Meanwhile, these mediators enter the articular cavity through blood circulation, aggravating synovial inflammation and cartilage matrix degradation (Hu et al., 2011; Kahn et al., 2022). In addition, increased intermuscular adipose tissue alters the mechanical properties of muscles, impairs their capacity to buffer mechanical loading, and further elevates abnormal stress on the knee joint, forming a vicious cycle of fat infiltration, inflammation and cartilage damage. Compared with muscle quantity indicators, intermuscular adipose tissue content is more sensitive and stable, suggesting that it may be a potential candidate biomarker for KOA risk stratification. However, its predictive value for incident KOA needs to be further verified by large-scale prospective cohort studies.

This meta-analysis systematically verified the close relationship between declined muscle strength and KOA development. KOA patients presented significant reductions in knee extensor torque, extensor strength, flexor torque and flexor strength. Low knee extensor strength was associated with higher odds of KOA (OR = 1.45, *P* = 0.01), which aligns with the fundamental role of muscles in maintaining knee dynamic stability (Yazdi et al., 2016; Espinosa et al., 2020). Furthermore, a prominent gender difference was observed in the subgroup analysis: low extensor strength significantly elevated KOA risk only in females (OR = 1.54, *P* = 0.0006), whereas no significant association was found in males. This suggests that females should be regarded as the priority population for muscle-targeted intervention in KOA, and early extensor strength training may substantially reduce their disease risk. Given the limited number of included studies, this sex-specific difference should be interpreted as preliminary and requires further verification in large-scale cohort studies. Compared with well-established KOA risk factors for knee osteoarthritis (KOA) such as obesity (BMI ≥ 24 kg/m², with a typical OR of 1.3) and trauma history (OR = 1.37) (Dong et al., 2023), the magnitude of the association between low knee extensor strength and KOA is comparable, with an OR of 1.45.

Although reliable conclusions were drawn based on rigorous systematic review methodologies, several limitations of this study should be acknowledged. (i) Moderate to high heterogeneity existed in several outcomes, particularly for knee extensor torque (I²=97%), knee flexor torque (I²=99%) and intermuscular adipose tissue (I²=72%). Heterogeneity may mainly originate from inconsistent measurement modalities (MRI, CT or ultrasound) and non-uniform standardization protocols across included studies. (ii) Most included studies were cross-sectional and case-control designs, with only six cohort studies included. Therefore, this review can only demonstrate associations between muscle parameters and KOA, and definitive causal relationships cannot be established. (iii) Several outcomes were based on a small number of eligible studies; for instance, indicators such as sartorius CSA and vastus lateralis thickness only included 2–3 studies, and the stability and generalizability of these results require further verification. (iv) Publication bias could not be fully excluded. Funnel plot analysis was only performed for knee extensor torque due to the small number of studies for other outcomes, and potential publication bias cannot be ruled out for outcomes with insufficient study numbers. (v) Residual confounding cannot be excluded across observational studies. Although adjusted effect estimates were preferentially extracted, the confounding factors controlled in each study were inconsistent, and the independent influence of BMI, physical activity, age, sex, and KOA severity on the observed associations cannot be fully ruled out.

## Conclusion

This systematic review and meta-analysis comprehensively evaluated the associations of thigh muscle quantity, quality and strength with KOA. The results showed that reduced vastus lateralis thickness, increased intermuscular adipose tissue content, and declined knee extensor and flexor strength were significantly associated with KOA. Among them, the association between low knee extensor strength and KOA appeared to be more prominent in women, which requires further verification. The compensatory enlargement of sartorius and flexor muscle CSA may be an early feature of muscle function imbalance in KOA. Muscle parameters, especially intermuscular adipose tissue and knee extensor strength, have potential value for KOA risk stratification. However, current evidence is mainly based on observational studies, and it is premature to incorporate them into routine clinical screening due to lack of evidence on feasibility, cost-effectiveness, and predictive accuracy. Future large-scale multicenter prospective cohort studies are needed to clarify the causal relationship between muscle abnormalities and KOA, and to verify the clinical application value of muscle-based assessment strategies.

## Data Availability

Data supporting the findings of this study can be found in the article. The protocol of this systematic review and meta-analysis is available in the Prospective Register of Systematic Reviews (PROSPERO) under the number CRD420261350323.
