# Supplementary material for: Association between imbalanced lower extremity muscle quality and the development of knee osteoarthritis: a Systematic Review and meta-analysis
Source: Front Physiol. 2026 Aug 10;17:1886678. doi: 10.3389/fphys.2026.1886678 (PMC13500577; doi:10.3389/fphys.2026.1886678)
Supplement: Supplementary file 1 [file DataSheet1.pdf]

Table 1 Search strategy used in PubMed database. 1442

No.	Query	Results
1	Osteoarthritis, Knee.ti, ab.	/
2	Knee Osteoarthritis. ti, ab.	/
3	KOA. ti, ab.	/
4	or 1-3	23,261
5	Muscle, Skeletal. ti, ab.	/
6	muscle mass. ti, ab.	/
7	skeletal muscle. ti, ab.	/
8	muscle cross sectional area. ti, ab.	/
9	muscle CSA. ti, ab.	/
10	intramuscular adipose tissue. ti, ab.	/
11	muscle volume. ti, ab.	/
12	quadriceps femoris muscle. ti, ab.	/
13	hamstring muscle. ti, ab.	/
14	rectus femoris muscle. ti, ab.	/
15	vastus medialis muscle. ti, ab.	/
16	vastus lateralis muscle. ti, ab.	/
17	vastus intermedius muscle. ti, ab.	/
18	biceps femoris muscle. ti, ab.	/
19	semitendinous muscle. ti, ab.	/
20	semimembranosus muscle. ti, ab.	/
21	extensor muscle. ti, ab.	/
22	flexor muscle. ti, ab.	/
23	thigh muscle. ti, ab.	/
24	or 5-23	205,637
25	4 and 24	1,442

Table 2 Search strategy used in Embase database.803

No.	Query	Results	Date
#23	#3 AND #22	803	25-Mar-26
#22	#4 OR #5 OR #6 OR #7 OR #8 OR #9 OR #10 OR #11 OR #12 OR #13 OR #14 OR #15 OR #16 OR #17 OR #18 OR #19 OR #20 OR #21	219,524	25-Mar-26
#21	'thigh muscle':ab,ti	3,778	25-Mar-26
#20	'extensor muscle':ab,ti	2,505	25-Mar-26
#19	'flexor muscle':ab,ti	1,932	25-Mar-26
#18	'semimembranosus muscle':ab,ti	307	25-Mar-26
#17	'semitendinous muscle':ab,ti	46	25-Mar-26
#16	'biceps femoris muscle':ab,ti	728	25-Mar-26
#15	'vastus intermedius muscle':ab,ti	102	25-Mar-26
#14	'vastus lateralis muscle':ab,ti	3,612	25-Mar-26
#13	'vastus medialis muscle':ab,ti	445	25-Mar-26
#12	'rectus femoris muscle':ab,ti	1,268	25-Mar-26

#11	'hamstring muscle':ab,ti	1,622	25-Mar-26
#10	'quadriceps femoris muscle':ab,ti	1,445	25-Mar-26
#9	'muscle volume':ab,ti	4,161	25-Mar-26
#8	'intramuscular adipose tissue':ab,ti	694	25-Mar-26
#7	'muscle csa':ab,ti	912	25-Mar-26
#6	'muscle cross sectional area':ab,ti	2,535	25-Mar-26
#5	'muscle mass':ab,ti	50,624	25-Mar-26
#4	'skeletal muscle':ab,ti	173,261	25-Mar-26
#3	#1 OR #2	34,676	25-Mar-26
#2	'koa':ab,ti	5,208	25-Mar-26
#1	'knee osteoarthritis':ab,ti	34,221	25-Mar-26

Table 3 Search strategy used in OVID database. 119

No.	Query	Results
1	Osteoarthritis, Knee.ti,ab.	1804
2	Knee Osteoarthritis.ti,ab.	7333
3	KOA.ti,ab.	519
4	1 or 2 or 3	8780
5	Muscle, Skeletal.ti,ab.	161
6	muscle mass.ti,ab.	7058
7	skeletal muscle.ti,ab.	29994
8	muscle cross sectional area.ti,ab.	547
9	muscle CSA.ti,ab.	169
10	intramuscular adipose tissue.ti,ab.	85
11	muscle volume.ti,ab.	788
12	quadriceps femoris muscle.ti,ab.	215
13	hamstring muscle.ti,ab.	422
14	rectus femoris muscle.ti,ab.	233
15	vastus medialis muscle.ti,ab.	79
16	vastus lateralis muscle.ti,ab.	725
17	vastus intermedius muscle.ti,ab.	13
18	biceps femoris muscle.ti,ab.	131
19	semitendinous muscle.ti,ab.	5
20	semimembranosus muscle.ti,ab.	31
21	extensor muscle.ti,ab.	390
22	flexor muscle.ti,ab.	301
23	thigh muscle.ti,ab.	689
24	5 or 6 or 7 or 8 or 9 or 10 or 11 or 12 or 13 or 14 or 15 or 16 or 17 or 18 or 19 or 20 or 21 or 22 or 23	37866
25	4 and 24	119
