# Supplementary material for: Association between imbalanced lower extremity muscle quality and the development of knee osteoarthritis: a Systematic Review and meta-analysis
Source: Front Physiol. 2026 Aug 10;17:1886678. doi: 10.3389/fphys.2026.1886678 (PMC13500577; doi:10.3389/fphys.2026.1886678)
Supplement: Supplementary file 2 [file DataSheet2.pdf]

Author(s):
Question: Thigh Muscle Quantity, Quality, and Strength compared to in KOA
Setting:
Bibliography:

Certainty assessment							№ of patients		Effect		Certainty	Importance
**№ of studies**	**Study design**	**Risk of bias**	**Inconsistency**	**Indirectness**	**Imprecision**	**Other considerations**	**Thigh Muscle Quantity, Quality, and Strength**		**Relative (95% CI)**	**Absolute (95% CI)**		
**Rectus Femoris Cross-sectional area**												
3	non-randomised studies	serious[a]	not serious	not serious	not serious	all plausible residual confounding would reduce the demonstrated effect	95 cases 95 controls<br>-	 0.0%	**OR 0.04** (-0.25 to 0.32)	-<br>**0 fewer per 1,000** (from 0 fewer to 0 fewer)	⨁⨁◯◯ Low[a]	NOT IMPORTANT
**Vastus Lateralis Cross-sectional area**												
4	non-randomised studies	serious[a]	not serious	not serious	not serious	all plausible residual confounding would reduce the demonstrated effect	110 cases 110 controls<br>-	 0.0%	**OR -0.04** (-0.31 to 0.22)	-<br>**0 fewer per 1,000** (from 0 fewer to 0 fewer)	⨁⨁◯◯ Low[a]	NOT IMPORTANT
**Vastus Medialis Cross-sectional area**												
3	non-randomised studies	serious[b]	not serious	not serious	not serious	none	92 cases 92 controls 0/0 exposed 0/0 unexposed<br>-	 0.0%	**OR -0.30** (-0.75 to 0.16)	-<br>**0 fewer per 1,000** (from 0 fewer to 0 fewer)	⨁◯◯◯ Very low[b]	NOT IMPORTANT
**Sartorius Cross-sectional area**												
3	non-randomised studies	serious[a]	not serious	not serious	not serious	none	2357 cases 2357 controls 0/0 exposed 0/0 unexposed<br>-	 0.0%	**OR 0.09** (0.13 to 0.15)	-<br>**0 fewer per 1,000** (from 0 fewer to 0 fewer)	⨁◯◯◯ Very low[a]	CRITICAL
**Adductor Cross-sectional area**												
4	non-randomised studies	serious[a]	not serious	not serious	not serious	all plausible residual confounding would reduce the demonstrated effect	2422 cases 2418 controls 0/0 exposed 0/0 unexposed<br>-	 0.0%	**OR 0.01** (-0.05 to 0.06)	-<br>**0 fewer per 1,000** (from 0 fewer to 0 fewer)	⨁⨁◯◯ Low[a]	NOT IMPORTANT
**Total thigh muscle Cross-sectional area**												
4	non-randomised studies	serious[c]	not serious	not serious	not serious	publication bias strongly suspected[a]	2668	2697	-	SMD **0.08 SD higher** (0.19 lower to 0.35 higher)	⨁◯◯◯ Very low[a,c]	NOT IMPORTANT
**Quadriceps Femoris Cross-sectional area**												
3	non-randomised studies	serious[c]	not serious	not serious	not serious	publication bias strongly suspected[a]	2463 cases 2706 controls 0/0 exposed 0/0 unexposed<br>-	 0.0%	**OR 0.03** (-0.14 to 0.20)	-<br>**0 fewer per 1,000** (from 0 fewer to 0 fewer)	⨁◯◯◯ Very low[a,c]	NOT IMPORTANT
**Flexors Cross-sectional area**												
4	non-randomised studies	not serious	not serious	not serious	not serious	none	2505 cases 2526 controls 0/0 exposed 0/0 unexposed		**OR 0.09** (0.04 to 0.15)	-	⨁⨁◯◯ Low	NOT IMPORTANT

							-	0.0%		**0 fewer per 1,000** (from 0 fewer to 0 fewer)		
**Extensors Cross-sectional area**												
3	non-randomised studies	serious[c]	not serious	not serious	not serious	publication bias strongly suspected[a]	188 cases 209 controls 0/0 exposed 0/0 unexposed		**OR -0.04** (-0.33 to 0.40)	-	⨁◯◯◯ Very low[a,c]	NOT IMPORTANT
							-	0.0%		**0 fewer per 1,000** (from 0 fewer to 0 fewer)		
**Quadriceps Femoris volume**												
4	non-randomised studies	serious[c]	not serious	not serious	not serious	publication bias strongly suspected[a]	153 cases 148 controls 0/0 exposed 0/0 unexposed		**OR 0.37** (-0.32 to 1.06)	-	⨁◯◯◯ Very low[a,c]	IMPORTANT
							-	0.0%		**0 fewer per 1,000** (from 0 fewer to 0 fewer)		
**Vastus Lateralis thickness**												
3	non-randomised studies	serious[c]	not serious	not serious	not serious	none	53 cases 63 controls 0/0 exposed 0/0 unexposed		**OR -0.61** (-1.18 to -0.05)	-	⨁◯◯◯ Very low[c]	CRITICAL
							-	0.0%		**0 fewer per 1,000** (from 0 fewer to 0 fewer)		
**Intermuscular adipose tissue**												
7	non-randomised studies	serious[c]	not serious	not serious	not serious	publication bias strongly suspected[a]	2672 cases 2856 controls 0/0 exposed 0/0 unexposed		**OR 0.35** (0.14 to 0.56)	-	⨁◯◯◯ Very low[a,c]	CRITICAL
							-	0.0%		**0 fewer per 1,000** (from 0 fewer to 0 fewer)		
**Knee extension torque**												
10	non-randomised studies	serious[c]	not serious	not serious	not serious	publication bias strongly suspected[a]	885 cases 1698 controls 0/0 exposed 0/0 unexposed		**OR -1.09** (-1.66 to -0.53)	-	⨁◯◯◯ Very low[a,c]	CRITICAL
							-	0.0%		**0 fewer per 1,000** (from 0 fewer to 0 fewer)		
**Extensor strength**												
6	non-randomised studies	serious[b]	not serious	not serious	not serious	publication bias strongly suspected[a]	840 cases 1892 controls 0/0 exposed 0/0 unexposed		**OR -0.16** (-0.24 to -0.08)	-	⨁◯◯◯ Very low[a,b]	IMPORTANT
							-	0.0%		**0 fewer per 1,000** (from 0 fewer to 0 fewer)		
**Knee flexion torque**												
4	non-randomised studies	serious[c]	not serious	not serious	not serious	publication bias strongly suspected[a]	668 cases 1511 controls 0/0 exposed 0/0 unexposed		**OR -0.95** (-1.88 to -0.02)	-	⨁◯◯◯ Very low[a,c]	IMPORTANT
							-	0.0%		**0 fewer per 1,000** (from 0 fewer to 0 fewer)		
**Knee extensor relative strength**												
4	non-randomised studies	not serious	not serious	not serious	not serious	none	307 cases 575 controls 0/0 exposed 0/0 unexposed		**OR -0.15** (-0.30 to -0.01)	-	⨁⨁◯◯ Low	CRITICAL

							-	0.0%		**0 fewer per 1,000** (from 0 fewer to 0 fewer)		
**Flexor strength**												
5	non-randomised studies	not serious	not serious	not serious	not serious	none	817 cases 1869 controls 0/0 exposed 0/0 unexposed		**OR -0.12** (-0.21 to -0.04)	-	⨁⨁◯◯ Low	IMPORTANT
							-	0.0%		**0 fewer per 1,000** (from 0 fewer to 0 fewer)		
**Knee flexor relative strength**												
3	non-randomised studies	not serious	not serious	not serious	not serious	none	216 cases 241 controls		**OR -0.18** (-0.37 to 0.00)	-	⨁⨁◯◯ Low	CRITICAL
							-	0.0%		**0 fewer per 1,000** (from -- to 0 fewer)		
**Low knee extensor strength**												
7	non-randomised studies	serious[c]	not serious	not serious	not serious	publication bias strongly suspected[a]	3192 cases 1202 controls 0/0 exposed 0/0 unexposed		**OR 1.45** (1.09 to 1.93)	-	⨁◯◯◯ Very low[a,c]	CRITICAL
							-	0.0%		**0 fewer per 1,000** (from 0 fewer to 0 fewer)		

CI: confidence interval; **OR:** odds ratio; **SMD:** standardised mean difference

Explanations

a. Most information is from studies at low or unclear risk of bias. Potential limitations are likely to lower confidence in the estimate of effect.
b. $I^2=50\%$
c. I^2[illegible]50%
