# Supplementary material for: Association between imbalanced lower extremity muscle quality and the development of knee osteoarthritis: a Systematic Review and meta-analysis
Source: Front Physiol. 2026 Aug 10;17:1886678. doi: 10.3389/fphys.2026.1886678 (PMC13500577; doi:10.3389/fphys.2026.1886678)
Supplement: Supplementary file 3 [file Table1.docx]

**Table S1 Quality Assessment of 11 Case Control Studies**

| Study | Selection | | | | Comparability | Exposure | | | Scores |
| --- | --- | --- | --- | --- | --- | --- | --- | --- | --- |
|  | Is the case definition adequate? | Representativeness of the cases | Selection of Controls | Definition of Controls | Comparability of cases and controls on the basis of the design or analysis | Ascertainment of exposure | Same method of ascertainment for cases and controls | Non-Response rate |  |
| Beattie KA[26] | ★ | ★ | ☆ | ★ | ☆☆ | ★ | ★ | ☆ | 5 |
| Culvenor AG[29] | ★ | ★ | ★ | ★ | ☆☆ | ★ | ★ | ☆ | 6 |
| Culvenor AG[30] | ★ | ★ | ★ | ★ | ★ | ★ | ★ | ☆ | 7 |
| Jasinevicius I O[20] | ★ | ★ | ★ | ★ | ☆☆ | ★ | ★ | ☆ | 6 |
| Kumar D[34] | ★ | ★ | ★ | ★ | ☆☆ | ☆ | ★ | ☆ | 5 |
| Maly MR[35] | ★ | ★ | ★ | ★ | ★ | ☆ | ★ | ☆ | 6 |
| Ruhdorfer AS[37] | ★ | ★ | ☆ | ★ | ☆☆ | ★ | ★ | ☆ | 5 |
| Taniguchi M[40] | ★ | ★ | ★ | ★ | ★ | ★ | ★ | ☆ | 7 |
| Taniguchi M[41] | ★ | ★ | ☆ | ★ | ☆☆ | ★ | ★ | ☆ | 5 |
| Watabe T[43] | ★ | ★ | ☆ | ★ | ★ | ★ | ★ | ☆ | 6 |
| Zhang X[46] | ★ | ★ | ★ | ★ | ☆☆ | ★ | ★ | ☆ | 6 |

**Table S2 Quality Assessment of 6 Cohort Studies**

| Study | Selection | | | | Comparability | Exposure | | | Scores |
| --- | --- | --- | --- | --- | --- | --- | --- | --- | --- |
|  | Representativeness of the exposed cohort | Selection of the non exposed cohort | Ascertainment of exposure | Demonstration that outcome of interest was not present at start of study | Comparability of cohorts on the basis of the design or analysis | Assessment of outcome | Was follow-up long enough for outcomes to occur | Adequacy of follow up of cohorts |  |
| Chin C[27] | ★ | ★ | ★ | ★ | ☆☆ | ★ | ☆ | ★ | 6 |
| Dannhauer T[31] | ★ | ★ | ★ | ☆ | ★ | ★ | ★ | ★ | 7 |
| Kemnitz J[33] | ★ | ★ | ★ | ★ | ★★ | ★ | ★ | ★ | 9 |
| Mohajer B[36] | ★ | ★ | ★ | ☆ | ★ | ★ | ★ | ★ | 7 |
| Segal NA[38] | ★ | ★ | ☆ | ☆ | ★ | ★ | ★ | ★ | 6 |
| Takagi S[39] | ★ | ★ | ★ | ★ | ☆☆ | ★ | ★ | ★ | 7 |

**Table S3 Quality Assessment of 7 Cross-sectional Studies**

| Study | Define the source of information (survey, record review). | List inclusion and exclusion criteria for exposed and unexposed subjects (cases and controls) or refer to previous publications. | Indicate time period used for identifying patients. | Indicate whether or not subjects were consecutive if not population-based. | Indicate if evaluators of subjective components of study were masked to other aspects of the participants. | Describe any assessments undertaken for quality assurance purposes (e.g.,test/retest of primary outcome measurements). | Explain any patient exclusions from analysis. | Describe how confounding was assessed and/or controlled. | If applicable, explain how missing data were handled in the analysis. | Summarize patient response rates and completeness of data collection. | Clarify what follow-up, if any, was expected and the percentage of patients for which incomplete data or follow-up was obtained. | Scores |
| --- | --- | --- | --- | --- | --- | --- | --- | --- | --- | --- | --- | --- |
| Aily JB[19] | 1 | 1 | 1 | 1 | 0 | 1 | 1 | 0 | 0 | 1 | 0 | 7 |
| Aily JB[25] | 1 | 1 | 1 | 1 | 0 | 0 | 0 | 0 | 0 | 1 | 0 | 5 |
| Conroy MB[28] | 1 | 1 | 0 | 1 | 0 | 1 | 0 | 1 | 0 | 1 | 0 | 6 |
| Hislop A[32] | 1 | 1 | 1 | 1 | 0 | 1 | 0 | 1 | 0 | 1 | 0 | 7 |
| Teoli A[42] | 1 | 1 | 1 | 1 | 1 | 0 | 1 | 0 | 0 | 1 | 0 | 7 |
| Yamauchi K[44] | 1 | 1 | 1 | 1 | 0 | 1 | 1 | 0 | 0 | 1 | 0 | 7 |
| Yamauchi K[45] | 1 | 1 | 1 | 1 | 0 | 1 | 0 | 0 | 0 | 1 | 0 | 6 |

**Table S4 Sensitivity Analysis in exclusion method**

| Indicators | Preliminary results | | | Exclude the study with the highest weighting | | | Exclude studies with the lowest weighting | | |
| --- | --- | --- | --- | --- | --- | --- | --- | --- | --- |
|  | *I^2^* | *SMD/OR* | *P* | *I^2^* | *SMD/OR* | *P* | *I^2^* | *SMD/OR* | *P* |
| Intermuscular adipose tissue | 72% | 0.35(0.14, 0.56) | 0.001 | 0% | 0.43(0.28, 0.57) | <0.00001 | 77% | 0.36(0.13,0.58) | 0.002 |
| Knee extensor torque | 97% | -1.09(-1.66,-0.53) | 0.0001 | 93% | -0.96(-1.43,-0.48) | < 0.0001 | 97% | -1.08(-1.68,-0.48) | 0.0004 |
| Knee flexion torque | 99% | -0.95(-1.88, -0.02) | 0.04 | 98% | -0.60(-1.57,0.37) | 0.22 | 99% | -1.08(-2.16,-0.01) | 0.05 |
| Low knee extensor strength | 64% | 1.45(1.09, 1.93) | 0.01 | 63% | 1.43(1.01,2.01) | 0.04 | 62% | 1.36(1.03, 1.78) | 0.03 |

**Table S5 Sensitivity Analysis in excluding lower-quality studies**

| Indicators | Preliminary results | | | Exclude the lower-quality study | | |
| --- | --- | --- | --- | --- | --- | --- |
|  | *I^2^* | *SMD/OR* | *P* | *I^2^* | *SMD/OR* | *P* |
| Intermuscular adipose tissue | 72% | 0.35(0.14, 0.56) | 0.001 | 74% | 0.34(0.11, 0.56) | 0.003 |
| Knee extensor torque | 97% | -1.09(-1.66,-0.53) | 0.0001 | 97% | -1.05(-1.67,0.42) | 0.001 |
| Knee flexion torque | 99% | -0.95(-1.88, -0.02) | 0.04 | 99% | -1.08(-2.16,-0.01) | 0.05 |
| Low knee extensor strength | 64% | 1.45(1.09, 1.93) | 0.01 | 69% | 1.63(1.03, 2.58) | 0.04 |
